# Genomic structural variation underlies cell type-specific betacyanin variegation in *Chenopodium quinoa*

**DOI:** 10.1007/s44154-025-00284-z

**Published:** 2026-02-11

**Authors:** Zheting Zhang, Yuwei Wang, Xiangwei Hu, Tiansheng Yu, Yaozu Feng, Jungao Zhang, Ting Zhang, Guojun Feng, Heng Zhang

**Affiliations:** 1 Shanghai Center for Plant Stress Biology, Center for Excellence in Molecular Plant Sciences, Chinese Academy of Sciences, Shanghai, 201602 China; 2 Department of Genetics and Developmental Science, School of Life Sciences and Biotechnology, Shanghai Jiao Tong University, Shanghai, China; 3 Current Address: Laboratory of Cell Fate Control, School of Life Sciences, Westlake University, Hangzhou, China; 4 Institute of Crop, Xinjiang Academy of Agricultural Sciences, Urumuqi, China; 5 Institute of Microbiology, Xinjiang Academy of Agricultural Sciences, Urumuqi, China; 6 Institute of Agricultural Resources and Environment, Xinjiang Academy of Agricultural Sciences, Urumqi, Xinjiang China; 7 Xinjiang Crop Chemical Control Engineering Technology Research Center, Urumqi, Xinjiang China; 8 Shanghai Pusha Investment Development Co., Ltd., Shanghai, China

**Keywords:** Quinoa, Variegation, Epidermal bladder cell (EBC), Betalain, Betacyanin, CYP76ADα, Structural variation, Subgenome, Gene cluster

## Abstract

The allotetraploid crop quinoa (*Chenopodium quinoa*) accumulates red/violet betacyanins, which function as vital stress-mitigating antioxidants. We investigated the genetic basis of red/green variegation observed in the aerial organs of the P0429 accession. We demonstrated that this color mosaic is primarily localized to epidermal bladder cells (EBCs), with red EBCs accumulating betacyanin levels ~ 50-fold higher than colorless EBCs. Cell-type-specific RNA-sequencing of EBCs identified the cytochrome P450 gene *Cqu0091301* (*CYP76ADα*) as the dominant and rate-limiting factor, exhibiting strong upregulation in red EBCs. This high pigmentation requires a specific structural variation in the P0429 accession: a ~ 4-kb genomic insertion that restores the full functionality of *Cqu0091301*, which is otherwise truncated and non-functional in common reference genomes. Genomic analysis reveals that *Cqu0091301* is part of a multicopy *CYP76ADα*–*DODA* gene cluster. Notably, expression analysis revealed functional divergence between the quinoa subgenomes, with B-subgenome *CYP76ADα* genes highly dominant in EBCs, while A-subgenome homologs were preferentially expressed in other tissues. Our results establish a clear link between structural genomic variation and cell-type-specific betalain biosynthesis, providing molecular insight into pigment regulation and subgenome specialization in allotetraploid quinoa.

**Supplementary Information:**

The online version contains supplementary material available at 10.1007/s44154-025-00284-z.

## Introduction

Variegation, defined as the occurrence of distinct color sectors within a single organ, is a widespread and important phenomenon in biology (Frank and Chitwood 2016). In plants, this mosaic coloration typically results from cellular differences in chloroplast development, pigment biosynthesis, or dynamic epigenetic regulation among neighboring cell lineages (Frank and Chitwood 2016). Furthermore, variegation can arise from genetic mosaics formed by spontaneous or induced mutations, as famously demonstrated by Barbara McClintock's work on spotted maize kernels (McClintock 1932). Transposon insertions and their epigenetic silencing have been linked to unstable expression of pigment genes in maize, morning glory, and other species, resulting in cell-type-specific coloration patterns (Iida et al. 2004). Consequently, variegated phenotypes serve as powerful systems for studying the mechanisms governing gene expression stability, epigenetic control, and cell lineage determination.

Quinoa (*Chenopodium quinoa* Willd.) is an ancient pseudocereal renowned for its exceptional nutritional profile and strong tolerance to various abiotic stresses, particularly salinity. As a facultative halophyte, quinoa does not require saline conditions for survival but is capable of completing the life cycle under 200 mM NaCl. A distinctive morphological feature of quinoa is the presence of specialized epidermal bladder cells (EBCs) covering most aerial organs (Shabala et al. 2014). EBCs are large, two-celled structures consisting of a stalk and a bladder cell, which is often considered the simplest form of salt glands (Dassanayake and Larkin 2017). EBCs have been reported to play critical roles in mitigating high salinity, enhancing herbivore resistance, and regulating osmotic pressure (Kiani-Pouya et al. 2017; Kobayashi and Fujita 2024; Miranda-Apodaca et al. 2025; Moog et al. 2023).

The red/violet pigmentation observed in quinoa is attributed to betalains, a class of water-soluble nitrogenous pigments characteristic of the order Caryophyllales (Timoneda et al. 2019). Betalains functionally replace the more ubiquitous anthocyanins in most members of this order. Betalains are divided into two major groups – the red/violet colored betacyanins and the yellow colored betaxanthins – both derived from the amino acid L-tyrosine. The core betalain biosynthetic pathway involves three primary enzymatic steps: (1) hydroxylation of tyrosine to L-DOPA by a cytochrome P450 enzyme (CYP76AD); (2) ring cleavage of L-DOPA by DOPA 4,5-dioxygenase (DODA) to form betalamic acid; (3) subsequent spontaneous condensation reactions leading to betacyanin and betaxanthin formation (Timoneda et al. 2019). Cytochrome P450 enzymes of the CYP76ADα lineage and DODAα isoforms play central roles in this pathway (Brockington et al. 2015; Polturak et al. 2016). Despite the identification of core enzymatic steps, the specific genomic drivers of intra-organ color variation and the potential for subgenome-specific regulation in allotetraploid species like quinoa remain largely unexplored. The contribution of genomic structural variations to betalain biosynthesis has not been reported. Beyond their role as natural colorants, betalain pigments are potent antioxidants, with accumulation frequently linked to increased tolerance to environmental stressors such as salinity, drought, and high light, primarily through the scavenging of reactive oxygen species and photoprotection.

Here, we investigate the genetic basis of a striking red and green mosaic variegated phenotype observed in the aerial organs of the quinoa accession P0429. We demonstrate that the color differences arise almost exclusively from the differential accumulation of betalains within the EBCs. Through cell-type-specific transcriptome analysis of isolated EBCs, we identified a key regulatory gene, *Cqu0091301*, a *CYP76ADα* homolog, whose expression strongly correlates with betacyanin accumulation in red EBCs. We further show that the reference genome annotation for this gene is incomplete; through genome resequencing and assembly, we discovered a ~ 4-kb genomic insertion in P0429 that restores a complete, functional P450 domain. Comprehensive genomic analysis revealed that *Cqu0091301* is part of a multicopy *CYP76ADα-DODA* gene cluster. Finally, we report an unbalanced contribution of the A and B subgenomes to betalain biosynthesis, with B-subgenome homologs showing preferential expression in EBCs and providing the dominant overall contribution across most pigmented organs. Our findings highlight a previously unrecognized role of structural genomic variation in controlling betalain biosynthesis and offer new insights into the cell-type-specific regulation of pigment formation and variegation in allotetraploid quinoa.

## Results and discussion

### EBC-specific betacyanin accumulation drives the unique red/green mosaic patterns

**Fig. 1:**
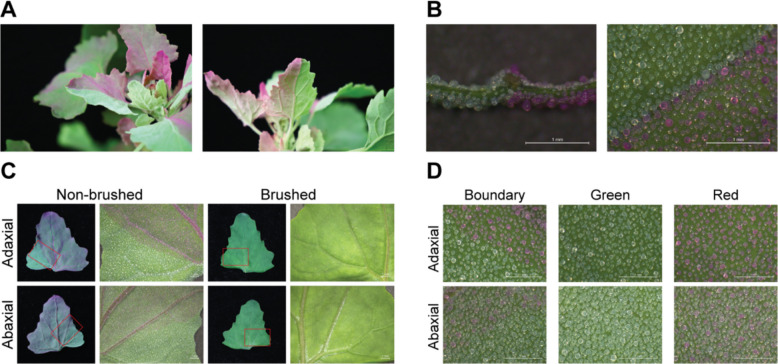
Variegation in the quinoa accession P0429. **A** The mosaic colored quinoa leaves. **B** Cross-section and top view of variegated leaves. **C** The adaxial and abaxial sides of the same leaf before and after EBC removal. **D** Boundaries of the mosaic colors

We identified several quinoa accessions within our germplasm collection that exhibit a striking variegated phenotype, characterized by distinct red and green sectors on aerial organs, particularly the leaves (Fig. 1A; Figure S1A, B). A cross-section of variegated leaves revealed that pigmentation is exclusively localized to the epidermal bladder cells (EBCs) in the red sectors, whereas the underlying leaf lamina tissue remained uniformly green (Fig. 1B). Mechanical removal of the EBC layer resulted in the originally red sectors becoming visually indistinguishable from the green parts of the leaf (Fig. 1C), indicating that variegation is caused by differential pigmentation in the EBC layer. The boundary between the two colored EBCs was sharp and developmentally stable, and occasionally aligned with the leaf vascular tissue (Fig. 1D). Given previous reports of betalain accumulation in quinoa (Otterbach et al. 2021), we hypothesized that the variegated phenotype results from differential betacyanin accumulation in EBCs.

**Fig. 2:**
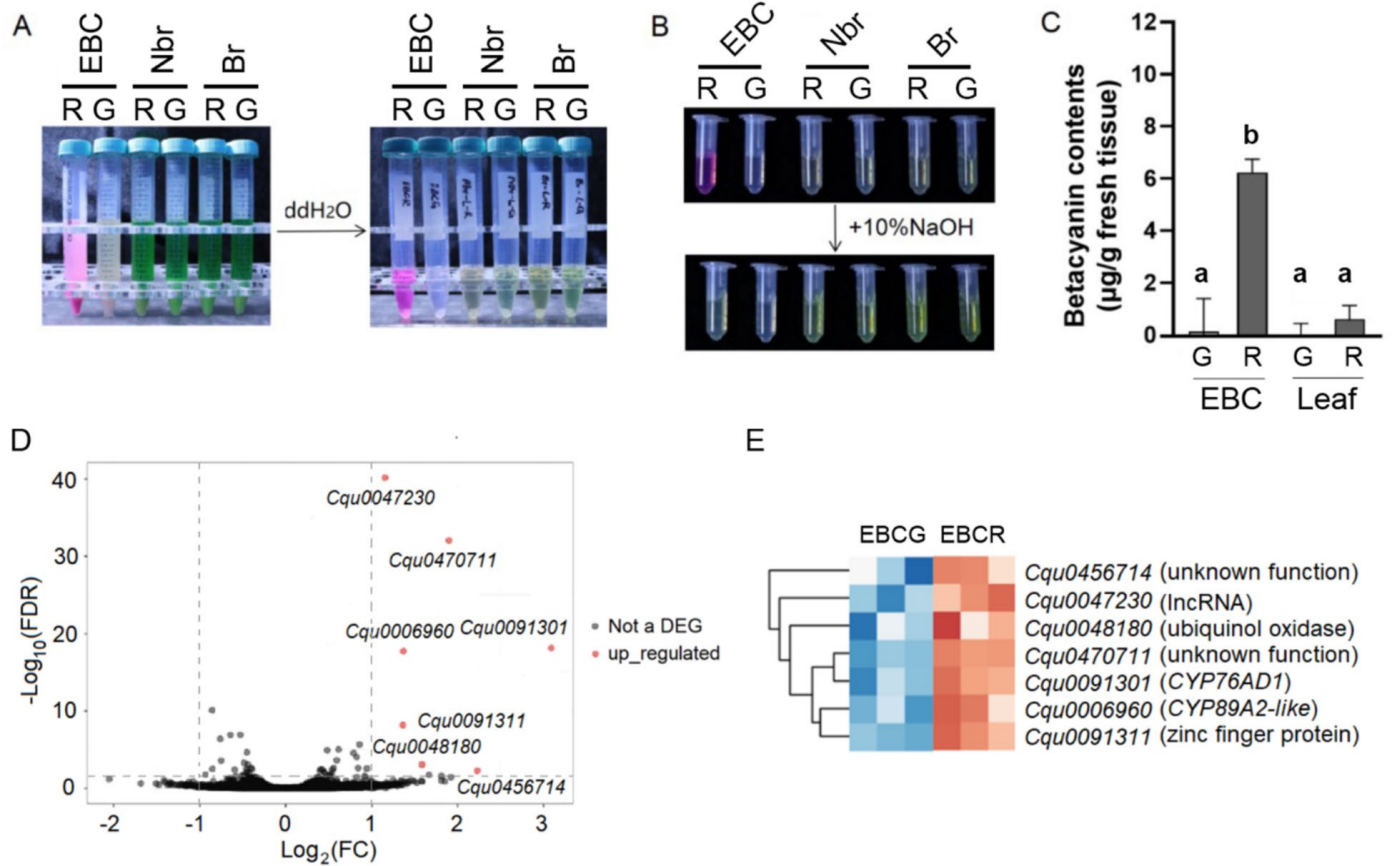
Betalain quantification and transcriptome analyses of EBCs. **A** Water soluble pigments from red (R) and green (G) sectors of variegated quinoa leaves (EBC: epidermal bladder cells; Nbr: non-brushed leaves; Br: brushed leaves without EBCs). **B** Sodium hydroxide reaction of betacyanins. **C** Quantification of betacyanin contents of the EBC and the leaf lamina in green (G) and red (R) sectors. **D** Volcano plot of the EBC transcriptome data. The transcriptome of EBCs in the red sector was compared to that of the green sector (control). **E** Heatmap showing the relative changes of the 7 DEGs identified (EBCG: EBC from green sector; EBCR: EBC from red sector)

To quantify this difference, we measured betacyanin content in isolated red and colorless EBCs using a standard protocol (Stintzing et al. 2003). Water-soluble extracts from red EBCs exhibited a strong purple-red coloration, while extracts from colorless EBCs were nearly transparent (Fig. 2A). Furthermore, the purple-red extract rapidly turned yellow upon the addition of sodium hydroxide, confirming the presence of betacyanins (Fig. 2B). Spectrophotometric analysis at the betacyanin absorption maximum (538 nm) revealed that red EBCs contained 6.23 µg/g betacyanins, which is approximately 50-fold higher than the 0.12 µg/g measured in colorless EBCs from green sectors (Fig. 2C). We also quantified betacyanins in leaf tissues after EBC removal. The red-sector lamina retained low levels (0.61 µg/g), while no detectable absorbance was measured in the green-sector lamina (Fig. 2C). Crucially, the difference in betacyanin content between the red and green EBCs was an order of magnitude larger than the difference observed between the underlying lamina tissues. In contrast, no detectable absorbance was observed in the green sectors (Fig. 2C). Moreover, we detected no significant differences in yellow betaxanthin levels (measured at 465 nm) between extracts from red and colorless EBCs (data not shown). Taken together, these results demonstrate that the leaf variegation phenotype is predominantly driven by the differential accumulation of betacyanins in the EBCs, with pigment differences in the underlying leaf lamina playing a minor role.

### Genes associated with differential betacyanin accumulation in EBCs

To identify the molecular mechanisms underlying differential betacyanin accumulation in epidermal bladder cells (EBCs), we performed RNA-seq on red and colorless EBCs isolated from the same variegated leaves of the accession P0429 (Figure S1C). Differential expression analysis identified only seven differentially expressed genes (DEGs), all upregulated in red EBCs (Fig. 2D; Table S1). The small number of DEGs was expected because EBCs were harvested from distinct sectors of the same leaf.

We next validated the expression level of seven DEGs using quantitative PCR (qPCR) in a new batch of EBC samples. Three genes (*Cqu0047230, Cqu0456714,* and *Cqu0470711*) were excluded from further analysis due to undetectable or unreliable expression (Ct values > 35). Of the remaining four genes (Figure S1D): (1) The P450 gene *Cqu0006960* (*CYP89A* family) showed low basal expression but was significantly upregulated in red EBCs. (2) *Cqu0091311* (zinc finger protein) was upregulated 4.5-fold. (3) *Cqu0048180* (ubiquinol oxidase) showed a statistically insignificant ~ twofold increase. (4) *Cqu0091301* showed both the highest absolute mRNA level and the largest fold change compared to colorless EBCs. Notably, *Cqu0091301* belongs to the *CYP76ADα*-type P450 family, which contains key components of the betalain biosynthetic pathway (Brockington et al. 2015). In contrast, *Cqu0006960* belongs to the *CYP89A* family, whose members are typically involved in leaf senescence (Mach 2013). Given its high expression level, large fold change, and strong association with betalain synthesis, we prioritized *Cqu0091301* for further mechanistic analysis.

### Structural variation of the *CYP76ADα* gene

**Fig. 3:**
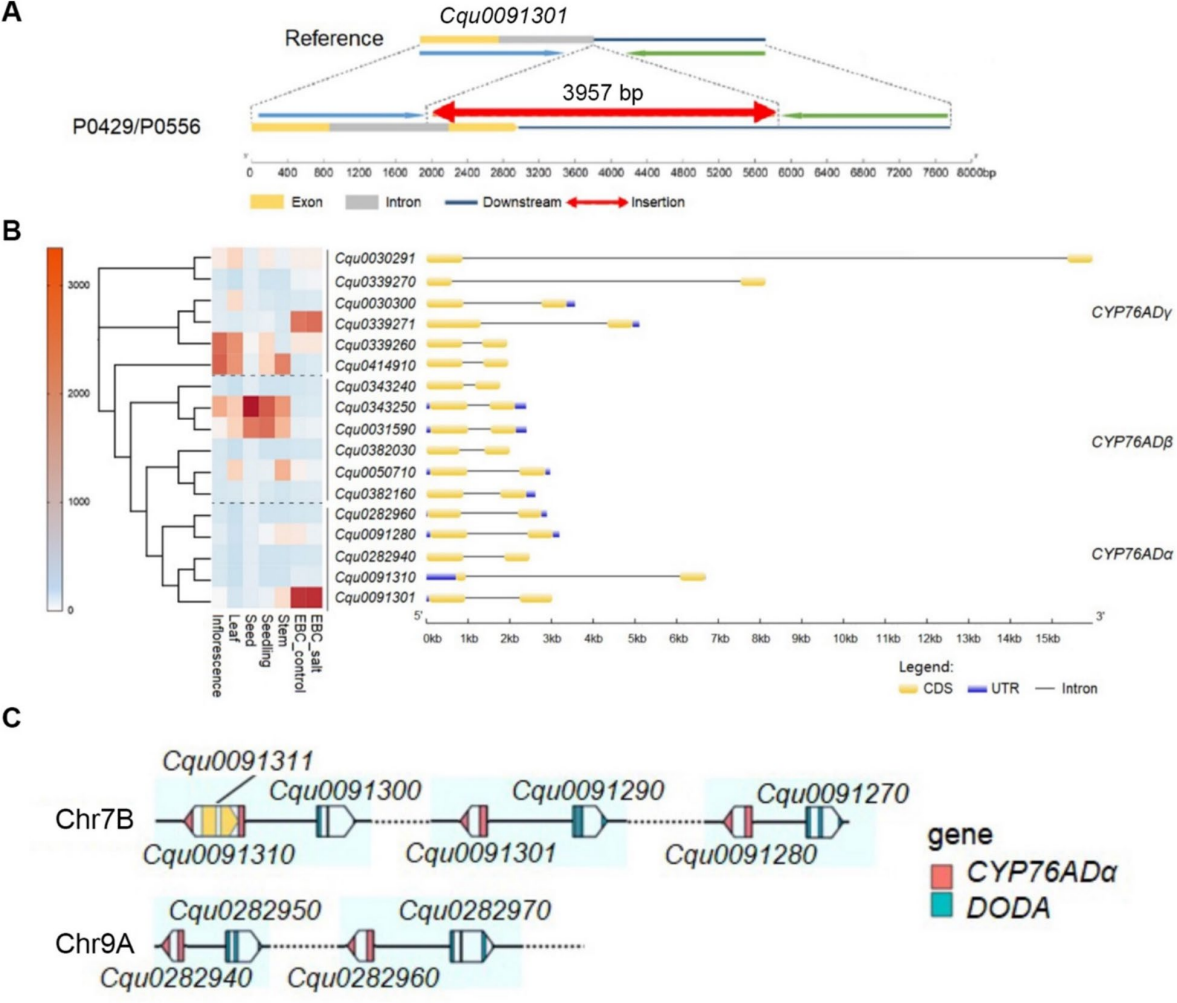
Genomic structure at the *CYP76ADα–DODA* gene cluster in quinoa. **A** Diagram showing the 3,957-bp genomic insertion that overlaps *Cqu0091301*. **B** Gene models and relative expression level of *CYP76AD* genes in quinoa. **C** Physical arrangement of the *CYP76ADα–DODA* gene clusters on Chr7B and Chr9A chromosomes of quinoa. Gene structures were not drawn to scale

Inspection of the gene models of *Cqu0091301* in Cq_real_v1.5 and previous reference genomes (Jarvis et al. 2017; Zou et al. 2017) revealed a truncated second exon, resulting in the loss of a conserved P450 domain (Fig. 3A). This structural anomaly suggested that the encoded enzyme was likely non-functional. However, RNA-seq data revealed a complex situation: (1) no reads aligned to the short second exon of *Cqu0091301* (Figure S2A); and (2) *Cqu0091280*, the closest homolog, showed a greater than tenfold enrichment of mismatched (SNP-containing) reads mapping specifically over a portion of its second exon compared to other regions (Figure S2B). We hypothesized that these mismatched reads originated from an unannotated, highly homologous transcript in accession P0429 that was absent or incorrectly modeled in the reference genome. This was confirmed by de novo transcriptome assembly, which recovered a transcript that perfectly matched the annotated *Cqu0091301* mRNA but contained an extended 3’ region (data not shown). These findings collectively suggest that the P0429 genome contains a genomic insertion at this locus, resulting in a full-length, more complete *Cqu0091301* gene compared to the reference.

To precisely map this genomic variation, we performed whole-genome resequencing of P0429. Visual inspection of alignment reads in IGV revealed numerous soft-clipped sequences aligning downstream of the *Cqu0091301* locus (Figure S3A). We performed de novo assembly but were unable to reconstruct the full insertion. However, we identified a 2,273-bp contig that matched the flanking sequence of the insertion site on one end. Using targeted PCR with one primer on this contig and the other on *Cqu0091301*, we successfully amplified and sequenced the full 3,957-bp insertion, which is missing in the reference genome but present in P0429 (Fig. 3A; Dataset S1). Re-annotation based on this new sequence confirmed that *Cqu0091301* in P0429 contains a significantly longer second exon, restoring the complete, conserved P450 domain.

Re-analysis of the RNA-seq data using the corrected *Cqu0091301* gene model resulted in a more pronounced expression difference between red and colorless EBCs (Figure S2C), and, conversely, a reduction in the apparent expression difference for the homolog *Cqu0091280* (Figure S2D). The expression patterns of the remaining DEGs were unaffected.

To contextualize the role of *Cqu0091301*, we further examined the expression of other betalain biosynthetic genes, including *TyDC* (Tyrosine decarboxylase), *DODA*, *cDOPA5GT* (cDOPA glucosyltransferase), *B5GT* (betanidin 5-glucosyltransferase), and *B6GT* (betanidin 5-glucosyltransferase) in EBCs. Most of these genes showed extremely low mRNA levels in EBCs (often an order of magnitude lower) and did not exhibit differential upregulation in red EBCs (Figure S4A). QRT-PCR analysis corroborated these patterns (Figure S4B). The only genes consistently and markedly upregulated in red EBCs were the three CYP76ADα genes: *Cqu0091301*, *Cqu0091280*, and *Cqu0091310* (Fig. S4B). Taken together, these expression data strongly indicate that the upregulation of the CYP76ADα genes, particularly *Cqu0091301*, is the key regulatory step driving betacyanin synthesis in red EBCs.

To understand the genomic context of *Cqu0091301*, we identified 17 members of the *CYP76AD* family in the quinoa genome based on sequence homology. These genes are grouped into three subfamilies (α, β, and γ), each containing 5–6 members (Fig. 3B; Figure S5). All the *CYP76AD* genes contain 2 exons, but their intron sizes are highly variable, ranging from 0.3 ~ 14 kb (Fig. 3B). All five *CYP76ADα* genes are physically linked and immediately adjacent to a *DODA* gene, forming *CYP76ADα–DODA* gene pairs located on two homologous chromosomes (Fig. 3C). Gene clustering is frequently observed in secondary metabolic pathways (Boycheva et al. 2014) and is often associated with the coordinated regulation of enzyme production, conferring evolutionary advantages and enhanced environmental adaptability (Zhan et al. 2022).

### Unbalanced contribution of the A and B subgenomes to betalain biosynthesis

**Fig. 4:**
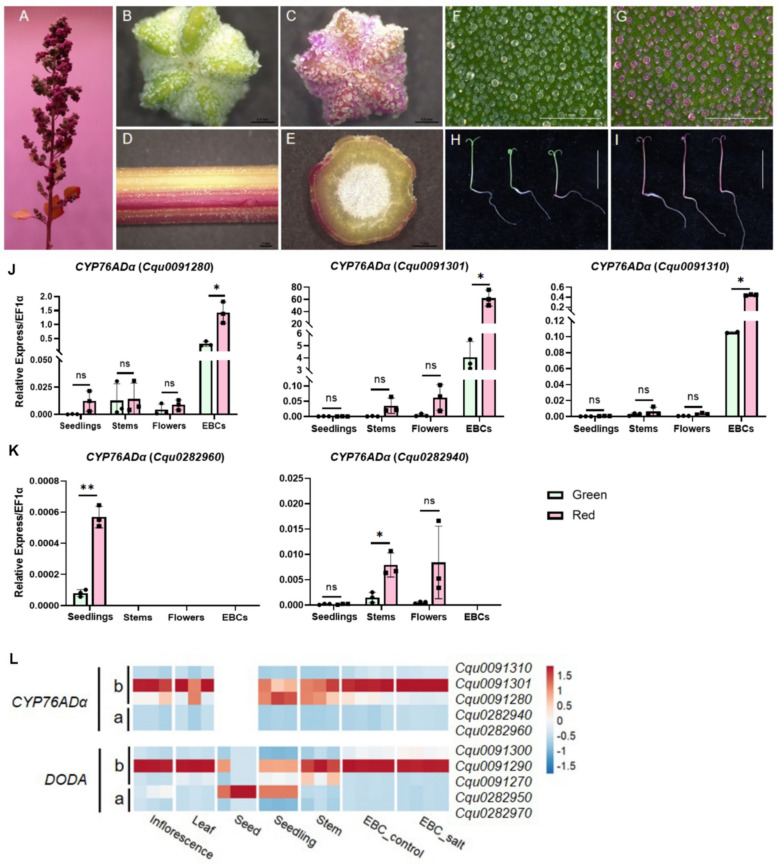
Expression of *CYP76ADα* genes in different quinoa organs*.* **A-I** Morphology of different organs of P0429 with varying colors, including inflorescence (**A**), flowers (**B**, **C**), stems (**D**, **E**), epidermal bladder cells (**F**, **G**), and hypocotyls (**H**, **I**). **J** and **K** The mRNA expression level of B-subgenome (**J**) and A-subgenome (K) *CYP76ADα* copies in green and sectors of various tissues. **L** Heatmap based on published transcriptome data showing the relative mRNA levels of *CYP76ADα* and *DODA* genes within each specific type of tissue

Beyond EBCs, the P0429 accession also exhibited distinct pigmentation patterns across various organs, including sharply segregated red and green sectors in the inflorescence (Fig. 4A–C), red epidermal stripes on the mature stem (Fig. 4D, E), and red pigmentation in the hypocotyl and cotyledons of seedlings (Fig. 4H, I). We therefore investigated whether the *CYP76ADα* genes identified as critical in EBCs also regulate pigmentation in other tissues. Using qRT-PCR, we examined the mRNA level of the five *CYP76ADα* genes in green- and red-colored sectors of seedlings, stems, and flowers. Among these homologs, the three B-subgenome copies *Cqu0091280*, *Cqu0091301*, and *Cqu0091310* showed a strong preferential expression in EBCs (Fig. 4J). In contrast, the mRNAs of A-subgenome genes *Cqu0282960* and *Cqu0282940* were not detected in EBCs; *Cqu0282960* was preferentially expressed in seedlings, and *Cqu0282940* exhibited preferential expression in stems and flowers (Fig. 4K). The expression of both genes was significantly up-regulated in the red tissues (Fig. 4K). These results indicate that *CYP76ADα* genes from the A- and B-subgenomes have evolved distinct, non-overlapping tissue expression preferences.

To broadly assess the contribution of the *CYP76ADα*–*DODA* gene pairs, we examined our earlier quinoa transcriptome datasets in the Real variety (Zou et al. 2017). The expression patterns of *CYP76ADα* and *DODA* across tissues support the concept of transcriptional co-regulation within the gene clusters. While *CYP76ADα* genes are generally expressed at an order-of-magnitude higher basal level compared to *DODAs* across various tissues, their relative expression profiles remain highly similar (Fig. 4L), suggesting they are governed by common regulatory mechanisms.

In most tissues, except for dry seeds and seedlings, the B-subgenome genes *Cqu0091290* (DODA) and *Cqu0091301* (CYP76ADα) were the most highly expressed within their families (Fig. 4L). Since *Cqu0091301* in the Real variety is structurally incomplete (Fig. 3A), the primary functional contribution in these tissues (inflorescence, mature leaves, stems) is likely borne by the second-highest expressed B-subgenome pair (*Cqu0091270*-*Cqu0091280*). Thus, although the A-subgenome genes are preferentially expressed in non-EBC tissues, the B-subgenome *CYP76ADα* genes make a consistently greater overall contribution to betalain biosynthesis across most plant organs. This pattern clearly demonstrates functional divergence between the A and B subgenomes.

## Materials and methods

### Plant materials

The quinoa plants (accession P0429, also known as CHEN198) were grown in the growth room at the Chenshan Botanical Garden Research Center. Plants were cultivated for 40 days under a day-night cycle of 14–10 h and 28-22ºC, with a light intensity of 300 μmol·m^−2^·s^−1^. Leaves exhibiting the variegated phenotype were sampled from quinoa plants with consistent growth. For transcriptome analyses of EBCs, each biological replicate was harvested from five individual quinoa leaves.

### Extraction and measurement of betacyanins

Weigh 0.1 ~ 0.5 g of tissue (EBCs, leaf lamina, or whole leaf) from both the red and green sectors of the variegated quinoa phenotype. Grind the samples into a fine powder in a pre-chilled mortar. Immediately transfer the pulverized sample to 7 mL of pre-cooled methanol at 4ºC. Shake vigorously and incubate for 30 min at 4ºC. Centrifuge the mixture at 12,000 rpm for 10 min, discard the supernatant, and retain the precipitate. Resuspend the precipitate in 3 mL of double-distilled water. After incubating for 30 min at 4ºC, centrifuge again at 12,000 rpm for 10 min and collect the aqueous supernatant. Measure the absorbance value at 538 nm using a UV spectrophotometer.

### Library preparation and sequencing

Quinoa seeds were surface sterilized and grown in ½ MS medium supplemented with 0.7% agarose in a Percival tissue culture chamber. Genomic DNA was extracted from two-week-old seedlings using DNeasy Plant Kits (Qiagen). Epidermal bladder cells were brushed off the leaf surface with cosmetic brushes and immediately placed in liquid nitrogen. EBCs were homogenized on a TissueLyser II (Qiagen), and total RNAs were extracted from the EBCs using RNeasy Plant Mini Kit (Qiagen). The genome resequencing and transcriptome libraries were prepared at the Genomics Core Facility of Shanghai Center for Plant Stress Biology, Center for Excellence in Molecular Plant Sciences, following standard protocols, and sequenced on the Illumina NovaSeq platform in paired-end 150 bp sequencing mode.

### Transcriptome analysis

The raw reads were subjected to quality control and adapter trimming using trim_galore with a quality score threshold of 20. The processed clean reads were used for de novo assembly using the Trinity software with default parameters (Grabherr et al. 2011). Alternatively, hisat2 (v2.1.0) was used to align the clean reads to the quinoa Cq_real_v1.5 reference genome. Picard markdupulication was then used to remove PCR duplicates. The processed alignment files were used in featureCounts (v1.6.3) to count the number of high-quality, strand-specific, and uniquely mapped reads corresponding to each gene. The resulting read counts for each annotated gene were subjected to data reproducibility testing using the edgeR package in R. The TMM algorithm was applied for between-sample normalization, and FPKM values were calculated as the expression level for each gene. The batch information was included as a variable in the linear model during the calculation of differential gene expression significance using the generalized linear model. Genes with an adjusted p-value smaller than 0.01 and over twofold changes after fitting to the generalized linear regression model were defined as differentially expressed genes (DEGs).

### Reverse transcription and quantitative PCR

Total RNA was reverse transcribed into cDNA using the TransScript One-Step gDNA Removal and cDNA Synthesis Kit (TransGene, Beijing) for quantification of candidate gene expression levels. The mRNA component of the extracted quinoa EBC total RNA was used as the template for reverse transcription, performed according to the manufacturer’s instructions. Quantitative PCR was performed on a CFX96 Real-Time PCR system (Bio-Rad) using standard protocols, using primers listed in Table S2.

## Supplementary Information

Supplementary Material 1.

## Data Availability

The Cq_real_v1.5 genome assembly and annotation were deposited at CoGe (http://www.genomevolution.org/) with genome ID 69570.

## Abbreviations

EBC: Epidermal bladder cell
Br: Brushed
Nbr: Non-brushed
CYP76AD: Cytochrome P450 family 76AD
L-DOPA: L-3,4-dihydroxyphenylalanine
DODA: DOPA 4,5-dioxygenase
qPCR: Quantitative polymerase chain reaction
DEG: Differentially expressed genes
TyDC: Tyrosine decarboxylase
cDOPA5GT: CDOPA glucosyltransferase
B5GT: Betanidin 5-glucosyltransferase
B6GT: Betanidin 6-glucosyltransferase
