# Supplementary material for: Genomic structural variation underlies cell type-specific betacyanin variegation in Chenopodium quinoa
Source: Stress Biol. 2026 Feb 11;6(1):15. doi: 10.1007/s44154-025-00284-z (PMC12894481; doi:10.1007/s44154-025-00284-z)
Supplement: Supplementary file 1 — Supplementary Material 1. [file 44154_2025_284_MOESM1_ESM.pdf]

Figure S1

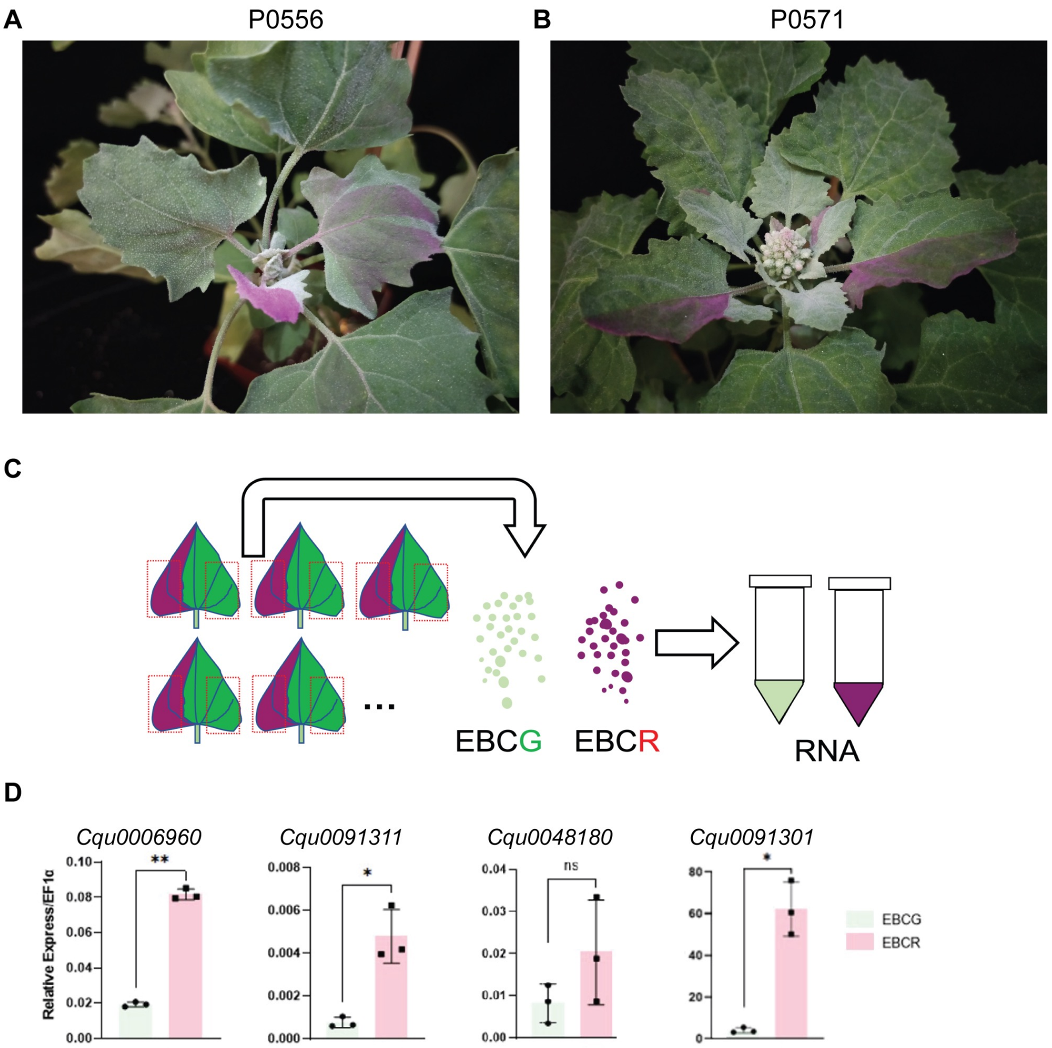

Figure S1 Variegation morphology and related gene expression analyses. (**A**) Leaf variegation in accession P0556. (**B**) Leaf variegation in accession P0571. (**C**) Schematic diagram of the experimental design of EBC-specific transcriptome analysis. (**D**) qPCR validation of DEGs identified in red (EBCR) vs green EBCs (EBCG).

Figure S2

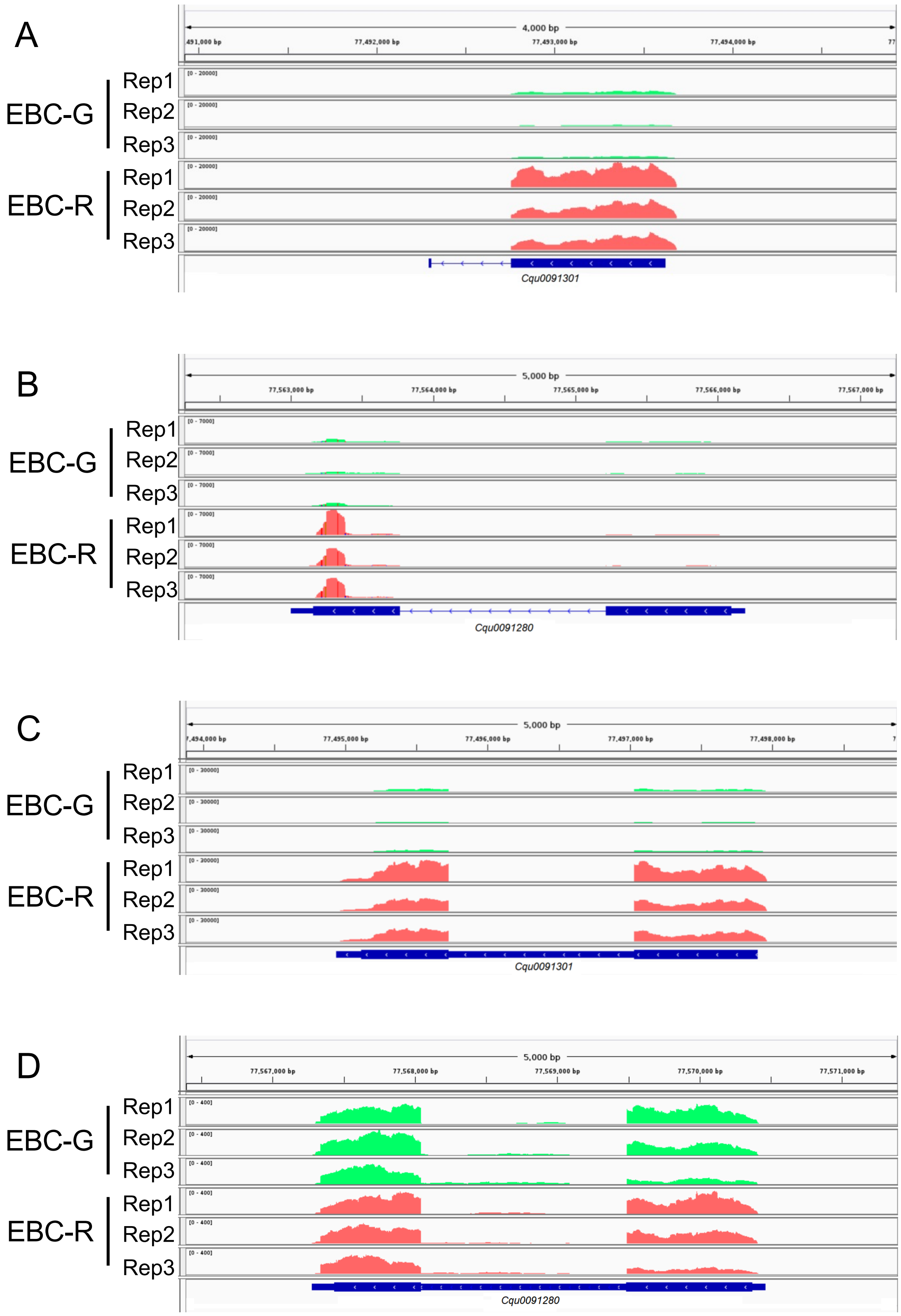

Figure S2 IGV (Integrative Genomics Viewer) snapshots of EBC transcriptome in P0429. (**A** and **B**) RNA-seq read coverage on *Cqu0091301* (A) and *Cqu0091280* (B) before the correction of *Cqu0091301* annotation. (**C** and **D**) RNA-seq read coverage on *Cqu0091301* (C) and *Cqu0091280* (D) after the correction of *Cqu0091301* annotation.

Figure S3

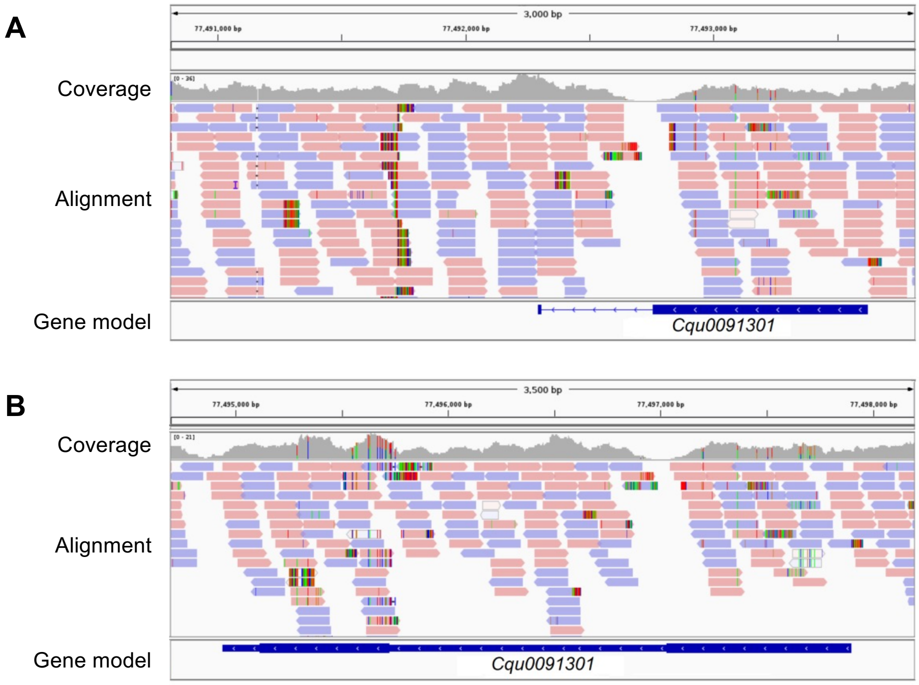

Figure S3 IGV (Integrative Genomics Viewer) snapshots of P0429 genome resequencing data. Coverage and read alignments on *Cqu0091301* before (**A**) and after (**B**) sequence correction.

Figure S4

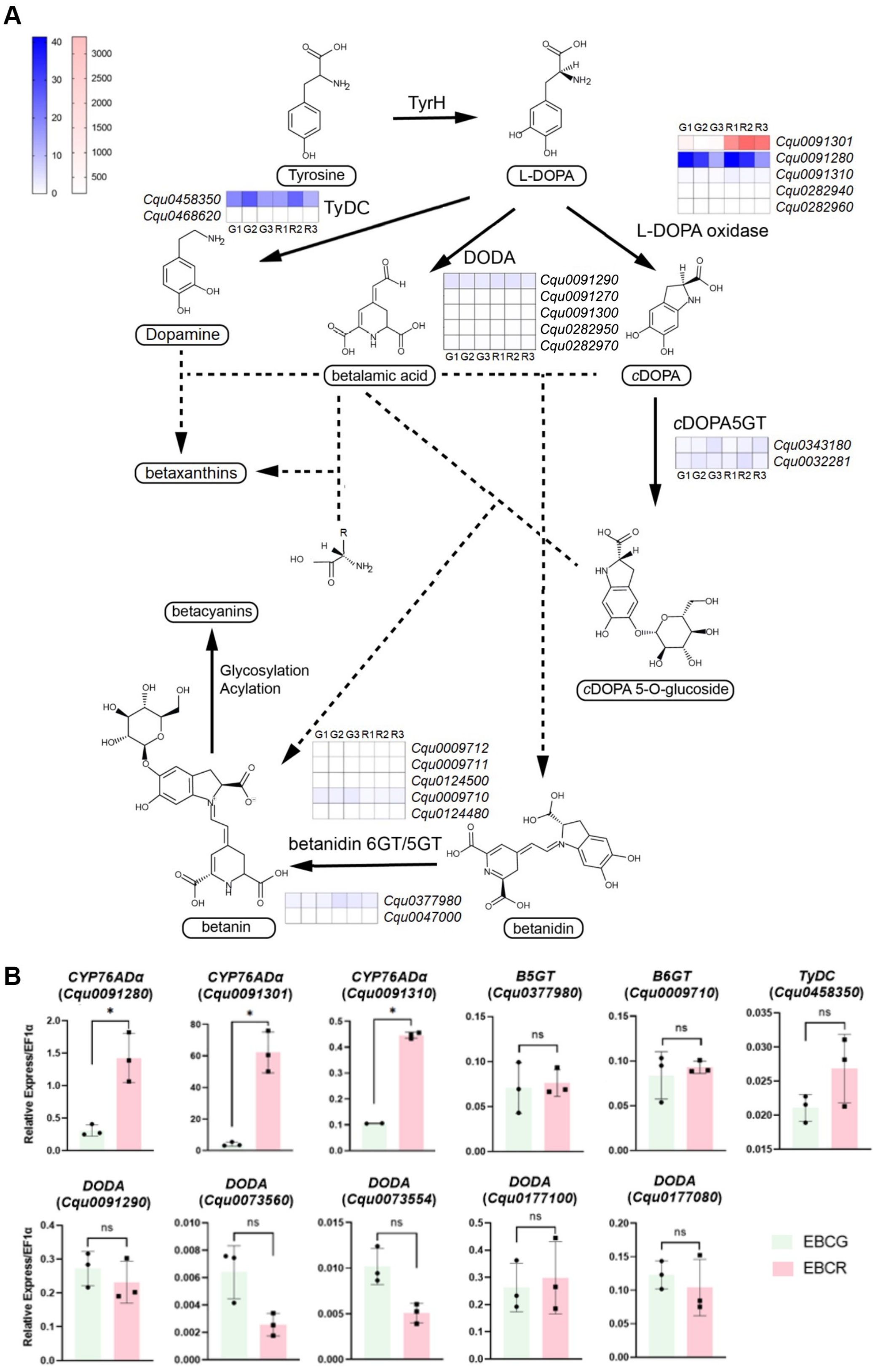

Figure S4 Expression of betalain biosynthetic genes. (**A**) Heatmap of the expression level of betalain biosynthetic genes in EBCs presented in a pathway. (**B**) qRT-PCR validation of betalain biosynthetic genes in green (EBCG) and red (EBCR) EBCs.

Figure S5

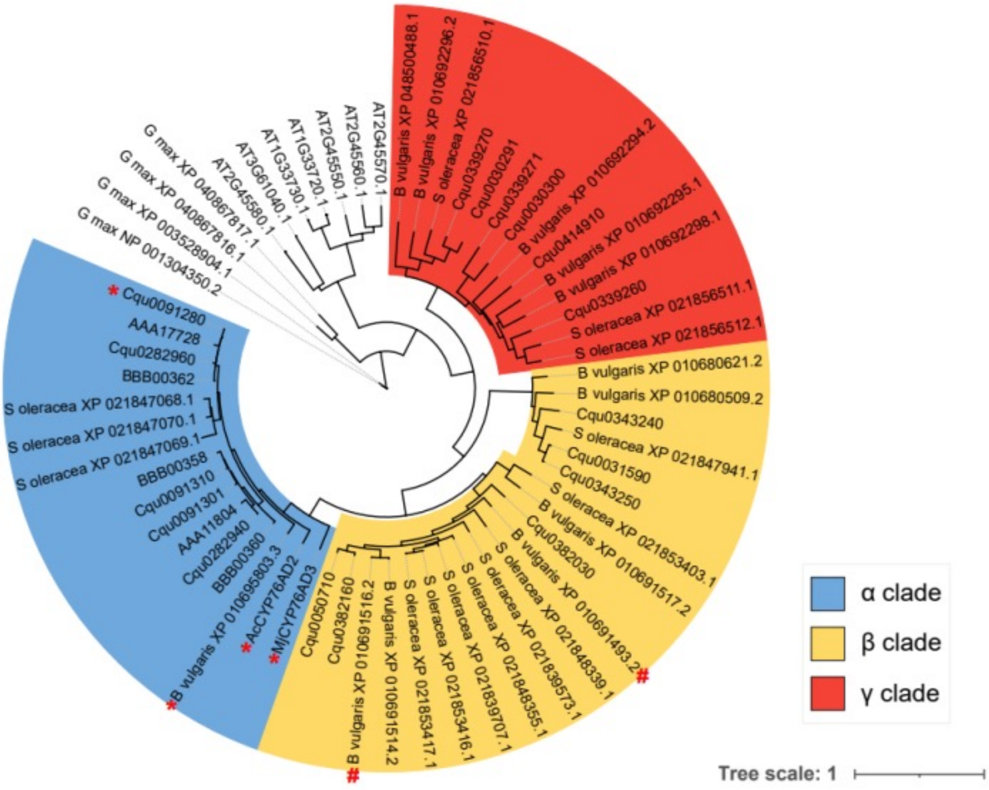

Figure S5 Phylogenetic tree showing the three clades of CYP76AD genes. *CYP76* genes from *Arabidopsis thaliana* and soybean were used as outgroups.

Table S1 Differentially expressed genes (DEGs) identified in P0429 EBCs

**Gene ID**	**NCBI ID**	**QQ74_v1 ID**	**EBCG_Rep1**	**EBCG_Rep2**	**EBCG_Rep3**	**EBCR_Rep1**	**EBCR_Rep2**	**EBCR_Rep3**	**p_value**	**logFC**	**Description**
Cqu0456714	LOC110696692	NA	0.33	0.76	0.12	2.10	2.15	1.09	6.94E-06	2.23	unknown function
Cqu0047230	LOC110718726;	AUR62010228	268.49	375.53	394.58	622.38	781.34	881.14	2.64E-45	1.15	lncRNA
Cqu0048180	LOC110718624	AUR62010135	2.38	0.95	1.68	7.05	3.12	4.70	7.16E-07	1.58	ubiquinol oxidase
Cqu0470711	LOC110685232	NA	7.80	6.30	7.64	27.46	25.97	26.90	6.97E-37	1.90	unknown function
Cqu0091301	LOC110733713	AUR62012348	725.70	284.81	499.04	5011.65	3677.58	3382.33	8.56E-23	3.09	CYP76AD1
Cqu0006960	LOC110681912	AUR62003503	12.43	9.92	8.64	30.36	29.79	19.68	2.95E-22	1.37	CYP89A2-like
Cqu0091311	LOC110733550	NA	4.76	5.53	4.78	14.04	13.12	11.44	1.92E-12	1.37	zinc finger protein

Table S2. Primers used for qRT-PCR analysis

Gene	Forward primer	Reverse primer
EF1α	GTACGCATGGGTGCTTGACAAACTC	ATCAGCCTGGGGGAGTACCAGTAAT
B5GT	AGAGATGGACAAGTTCTTTAAGGCT	AAGTTCTGATTCCTTGACTGCATTG
B6GT	GACTTTGGAGAGTTTGTGGTTCG	CCCTCACTTTGTTCTTCGTTTCC
DOPA5GT-1	ACCCAACACCGAAAATACCGATA	CTCCAGATTGAAACATACGCACC
DOPA5GT-2	TGTTTCAATCTGGACCAATCTACCT	ACAAACCTCTTCCTTCTCATCATCA
TyDC	GACGTAGCAAAGGAGTACAACATG	ACAACGTACAAGAGAGTTAGGGTC

```
1       10        20        30        40        50
|       |         |         |         |         |
ATGGATAATACAAGCCTAGCAATGATACTTGCAATTTGGTTCATTGCTTT
TCATTTCATTAAAATATTATTTACTAGCCAAACTTCCAAACTTCTTCCTC
CAGGCCCTAAACCACTTCCAATAATCGGCAACATTCTTGAAGTTGGTGAC
AAACCTCACCAGTCATTTGCTAACCTCGCCAAGATTCACGGCCCTCTAAT
ATCTCTACGTCTAGGCAGTGTCACAACTATTGTTGTATCATCAGCTGAAG
TAGCCAAAGAAATGTTCTTAAAAAAAGACTACCCTCTTTCTAACCGTACT
GTTCCTAATTCTGTCACTGCTGGTGACCACCACAAACTCACCATGTCGTG
GTTGCCTGTCTCCCCAAAGTGGAGGAATTTTAGAAAGATCACAGCCGTTC
ATTTACTTTCTCCTCAAAGACTTGATAGTTGCCAAAGCCTTAGGCATGCC
AAGGTACAACAACTTTTTCAATATGTACAAGAATGTGCACAAAAAGGGCA
AGCCGTTGATATTGGCAAGGCTGCATTTACTACATCCCTCAATTTGTTAT
CAAAACTATTCTTTTCGGTCGAATTAGCCCATCATAAATCCCATACATCT
CAACAATTCAAAGAACTTATATGGAATATTATGGAAGATATTGGCAAGCC
TAACTATGCTGATTACTTTCCAATCTTAGGATGTGTCGATCCTTCGGGTA
TTCGACGGCGATTAGCGTCTAGTTTTGACAAGCTAATTGCTGTTTTTCAA
AGTATAATCACTCAAAGGCTTGGTAGTACAACAACAAAGATAAATGATGT
GCTTGACGTTCTTCTCGACCTCTACAAACAGAAGGAGCTTAGCATGGCCG
AGATTAACCATCTTCTAGTCGTAAGTTCATTTTTTTTCTCCATATATATA
TATATATATATATATATATATATATATATATATATATATATATATATATA
TATATATATATATATATTCTAATGTAATCTTATGACTTTTGACTACCACA
ATTTTTTATAATGGATGGCTGAGATTATTTGTTTTTAGATTTATTTTTAA
TTAGTAATTTTATTTTTAAAAAAATCATTTTTAATTATTTTATATATATG
TTTTATTTCCTTAATTTTGAAAACATAAAAATAATTTCTGAAAGATTAAA
ATATATATTACGTATTTAAAATGATTAAAAATTAATTTTAATCAAATTTA
CGAATAAAAAATAAATTAAAAAATAAATAATCTCAGCCGTCCATTTTAAT
AAAATTGTGGTAGTCTAAAGTCATAAAACTACATTGAAATCATATATATG
TTTTCTTGTGTACGTAGTTTCGAGGAAATTGACGTAAATAGAATATCATA
TCAGACTAGTTCTTAGATTCACGTCAAAGATTTTGACGAATTAAGCTAAT
TACTACAATCATCAAGCATGCATCTACTAATTCTTAATATTTTTTCTTTA
CCAATAATGAGCTTGTGCATGGCATAAAATGATTTTTTAAGAAACACAAC
TTTACATTATAAAATTAATAACGAATTACGATTCATTGATTGGATATATA
TATATGTGACAAATATATAAACCACATTTATTTGGAGCTTGTTAAACTCT
GACCTGTCACATTAGATCAGAATTTAAAGTATACTTGTTACTGTTTTCAT
CTCAAATAATTGTTTTACTATACTATGCTTTGCAACAAAAACAAGTAAAA
TAGTATATTTGTTGATAAAAATTGTACGAGTAAGATTTTGATGTATATAA
TGAAAAAGGCAAAATAGTATGAGAAGATAGAACGCAACCCGTGCATTCAC
AATAATAAAACAAGAAAATTCCATTAAGAACAATTAAATGATTGGTAAGA
ACATTATTCTATAAAATAAAGAAAGTATACATTGTTTAATAGAATAATAG
ATACATTCTAGCTAAAAGTGGCGAACACAAAAAGAGCGATGTTTGTGATT
ATAAGAGTGTTAGACCTCACATTTTCTACGAAAAGTGCAAGGGTGCAAAT
GATTAGACCCCACAAATATCTACAAAGAAATTAATTAAAGAAAATTCATT
GATTAGGTTATATTGCATTCTTGTAATAATTGATAGAATAAGTAATAAAG
CTATTAAATTAATTATTGCCAACTTATTTCGGTTTAATTGCTTAAATTAA
TTTAGTATAATTTATGCATGTCAGGATATATTTGATGCCGGGACTGACAC
TACATCAAGTACTTTTGAATGGGCAATGGCAGAGTTAATTCGAAATCCTA
AAATGATGGACAAAGCTCAAAAAGAAATTGAGCAAGTCTTGGGCAAGGAT
AGACAAATTCAAGAATCAGACATTATTAAGTTACCTTACTTACAAGCCAT
TATCAAAGAAACATTGCGACTACACCCACCAACTGTATTTCTCTTGCCTC
GTAAAGCTAATTGTGATGTTGATTTATTTGGCTATGTTGTGCCAAAAGAT
GCACAAATACTTGTTAATTTATGGGCTATCGGTAGAGATCCTCAAGCATG
GGTGAACTCTGATGTGTTTTTACCTGAGAGGTTTTTGGGATCCGAAATTG
ATGTAAAGGGGAGAGATTTTGGACTCTTACCTTTTGGAGCTGGAAGGAGA
ATATGCCCAGGGATGAATTTGGCTATTAGAATGTTAACTTTGATGTTAGC
TACGCTTCTTCAATTCTTCAATTGGAAGCTTGAAGAAGGTATGAACCCAG
AAGATCTAGACATGGATGAAAAATTTGGAATTGCCTTACAAAAGACTAAA
CCTCTTCAGATCATTCCGGTTCTTAGGTATTATTGATCGTTGTCAAATGT
TTACGTATTTATATGTTTTTGTCAGTAAATTCATTCACTTTCTAATTGTT
TAAGTTTTCTACAAATGTTCATGTTTGTTTGAATCTTCCATTCAATGCAA
TTTTAAGGTGGTACTAGAGATCAAGTTTATGTACGTTAGCTCATATGCTA
```

TTTATTGTAATCTATGTTTACATATAAATTGTTAATAAAAGTTTGTCAAT
TGTTTGATGGATATTTTTGGTGATTTCATCAGCAGTTTTTTACTGTTCAT
AATACATGCTATTTAGGCCAAGTCTTCATAGCAAGCTAGTGTGAAACTTC
TATTCTAATCTCCTTGTTTTTTTAGGAAAATTTGACATTTGCTACCACCC
AAAACGCCTCGCTTTAGATTTGCTACCATTTTAATTTTTTTTTTACGTTGC
TACCACCTAATTCAAGTTTGTTAGAAATTACTACTACTTAACGGAATCCG
TTAGGATTTCCGTTAAGTTTATTAAAAAAAAAAGAAAAAGAAAAAAAACCC
AGCCACCCCACTTTGCCACTCTTTCCCCTCAACTGCCACCCCTCCCCCTG
CCGGCGGCCGGCGCCGGCGACCTCACCCCCCTCCCTTTTCCCCACCCACG
AAGGCAACCCTCCTCCCTTTTCCTCACCCATGAATCCAAATCTACCACCC
ACGAAGCCAACCCCCACCGAGATCAGCCCCCCTCCCCGTTACCCCACCGG
AATCGAACCCCTACCCTTCCTCCACCCAAAAACTGACACCCCTAACCCTA
AACCACCAGCTCGAAACCGCCTAAACGGGTTTTAGGGTTTCGCTTGTGGC
TGATTGGGGGGTGTTGCAAATTGTGGTGGCTGCAAATTGAGGAGGGTGCA
AATTAAGGTGGTTGCGAATTGAGGAGGCTGCAAAATTGGTGGCGGAGTTG
GTGTTGCAGGTGGGTGCGAATTGGTGGGTGCAAATTGAGGGTTTGCGAAA
TTGGTGGCTGAGTTGGTGGTTGCGAATTGGTGGTTGATTTGGTGGAGGGA
ACGGGGAGGGGGGGTTGAATACGGTAGGGGTTGGCTTCGGTGGGTGGGGG
AAAGGAAGGTGGGGGTTGGCTTCGTGGTGGGGGAAAGGGAAGGGGGTTAG
CTTCATGGGTGGGGGAAAGGGAGGGGGAGGGGTCGTCGGCGCCGACCGTA
GTTTTGGGCGCCGACCGCTGGTAGGAGGGAGGGGGAGGGGTGGCAATTGA
GGGGAGGGGGTGGCAGTGGGGTGGCTAGACGGTGGTACGGCCCCACTGAC
GGAAATTGTAACGGTGTAAGGCATGGGGTAGTAATTTCTAACAAACTCTG
TTTAGGGTGGTAACAAAGTAAAAAAAAAAAAATTAAACGGTGGCAAATCTG
AAGGTAGGTGTTTTAGGTGGTAGAAAATGTCAAAATTTCCTTTTTTTTAC
TAGCTAGTTCAAAGGAAATTGTAAAGAATTTTAGATGGAGACTTCTATCT
CTCGACACCACAACATGATTGTGACTTGGCTCAATGTTACTGAGAGGAAT
TAAGCTTCAACAGAGGTCCAATACAATAAGAAAAAGCCCCATCAAGTAAT
TTGACCTCATTTGGCATATTTTGTAGTCAATCTTAACTCAATTATTACTA
TGTCATATTTATGATAAAATATTAGCACAACTATTAGGGTAGCTTCAACC
TCTAGGAACTTAAGAGGCATGGCTCTAGCATAATTAAGTCCTTCCCTTAA
TGAATAGAGTTCCGCTCTTAGAGCATTAGGAGCATTATATTTTCTAGAAT
AGCCAAAATGCCACTCTCCTTTTGCATTACTAAAAGCACCACCTCCACCT
CCTAATTTTGTAGAAACCCAAGCACCATCTGTGTTTAATTTCAGAAACCC
TGGCTTTGGGGACGCCAGCTAATATCCACGCTTTTTAGAAATTTTGTAGG
ATTCAAGGTCTGGTGTCGAAAAGACTTGAGATTATAGAGGATACATCTCG
GGGTCATATAATTCCGTTTGGAACCATATATGATCATTGTATGTTGTCAT
AAATTTTCGTGGTAGGCTTGATTTAATGAAATCGGTGCAAGTATTTACCA
GTAAATTAGTCGTTTAAAGGCAAATTATATGGTTACACCCACCCAATTTG
ACTCCCGGAGGATATTAGGGACCATGTTAAACGTTTCGGCAATTTTTATT
TCCTGACGAATAAAAGTCAAAAATTCCAAAACTAAAGGCTAAAATACACG
CTTTTTGACAAATTTTAGAGGTATCAAGGCCCGATGTCGAAAATCCTTGA
AATTTTTGGGGATACATCTCGAGGTCATATAATTCCGTTTGAAACAATAT
ATGATCATTATATATTGTCATAAATGTTCGTGGTAGGCATGATTTAATGA
AATCGGTGCAAGTTAATTGTAAACCGGTAAATTGAACGTTTAAAGGGAAA
TTGGATGGTTACAACCACCCAATTAGACTCCCGGAGGATATTAGGGATCA
TGGTAAACATTTCGGCAATTTTTATTTTCGAGCAAAAAAAATGTCAAAAA
TTTCCAAAAATGAAGACTAATATCCACGATTTTTGAGAAATTTCGAAGGT
TTCGGGGCCCGGCGTCGAAAAGAAATGAGATTTTAGGGGATACATTGGGG
TCATATAATGTCGTTTGAAACCATAAATGACCATTATATGTTGTCATAAA
TGTTCGTGGTAGGCTTAGTTAATAAAGTCAGTGCAAGTTGAAAACTGGTA
AGTTGGTCGTTTAAAGAAAAAATTAGATGGTTCAACCCTAATAATTGGAC
TCCCGGAGGTTACTAAGGACCATTGTAAACATTTCGCCAATTTTTATTTT
TGGGCGAAAAAAGTCAAAAAATTCCAAAATTAAAGGCTAATATCCACGTT
TTTTAAGAAATTTTGAAGTTTTAGCGATCCGATGTCGAAAAGACTTGAGA
TTTTAGGTAATACATTTCGGGGTCATATAACGTCATTTGGAATCATATAT
GATAATCATTTGTTATCGTAAAAGCTCTTGGTAGGTTTGAGTTAATGAAA
TCGGTGCAAGTTGTAAACATGTAAATTGGCCTTTTAAAGACAAATTTGAT
GGTTCTACCCATCCAATTGGACTTCGGGAGGATATTACAGACCATGATAA
AAATTTCGACAATTTTTGTTTCCGCACGAAAAAAAAAGTCAAAAAATTCA
AAAATTGAACGCTAATATCCACCTTTTTGATAAATTTTGGAGCCTTTGGG

```
GGGTGTCTAAAAGTCTTGAAATTCTAGGGAATACATCTTAGGGTCATATA
ATGTCGTTTTGAAACATATGTGATCATTATTTTTGTCATAAATGTTCGTG
GTAGGCTAGAGTTAATAAATTCGGTGCCAGTTGTCAACTTGTAAATTGGT
CATTTAAAGGAAAATTGAATGGTTCCACCCACCCAATTGGACTCCCGGAG
GATATTAGGGACCATGTAAAACATTTCGGCAATTTTATTTTCGGGGCCCG
ATGTTGAAAAGAAATGAGATTTTAGGGGATACAACATCTCGGGGTCATAT
AATTCTGTTTGAAACCATAAATGACCATTATATGTTGTCATCAATATTCG
TGGTAGGCTTGAGTTAATGAAATCAGTGCAAGTTGTAAACCGGTAAATTG
GTCATTTAAAGAAAAAAAATTGGATGGTTCCAACTTAGTAATTGGACTCC
TGGAGGGTATTAAGTACCATGGTAAACATTTCACCAATTTTTATTTCTGG
GCGAAAAAAAGTTAAAAATTTCCAATATTGAAGGCTAATATCCACGTTTT
TTAAGAAATTTTGAAGTTTTCGGGACCTAGTGTCGAAAAGACTTGTTGTA
GACACCCAAATGTGTCTCCCCAAAACCAAACCCAGTGATGAGTACTTTTC
ACGTCGAAACATAATCGAATGAAGTGAAAGTGTGGAAAATCTAGTGTTGC
GGATAATTTTAATTTCTTTTCTCTTCTCTCTGTTTTTGAAAATTTATTTT
AAGAAAACAGCCAATCCAGTTTGGTTGGTCCATGATATGGGTCCAATATT
AGTGTAGTTGGATGGGGCTTGCAAAGACCTTTCCAACGATATATTATAAG
CCCAATTCCGATGAATATTGAGGAAGTTATGGCCAATTTACCAAACTGGG
CCCGGTTTGAGCTTTATAAACGGCCCAATGATCTAGGGGAGCGCCTAATA
TGATTTATCATTCCTAATGCAATTAGGAATGGTTTTAGTCTTCTAGATTC
```

Dataset S1 The genomic sequence of P0429 containing the complete *Cqu0091301* gene and the ~4-kb insertion. Note: the genomic sequence is in the same direction as the open reading frame of *Cqu0091301*.

Orange-colored letters: Exon sequence of *Cqu0091301*

Grey-shaded letters: the 3,957-bp insertion sequence in P0429 and P0556
